# Stakeholder needs assessment for developing ageing in place solutions – a qualitative study

**DOI:** 10.1186/s12877-024-04722-x

**Published:** 2024-01-29

**Authors:** Andrea Kastl, Yvonne Nadine Rauner, Sandra Mayer-Huber, Claudia Oestreich, Franz Benstetter, Ulrike Fettke

**Affiliations:** Technical University of Applied Sciences Rosenheim, Hochschulstraße 1, Rosenheim, 83024 Germany

**Keywords:** Stakeholder needs analysis, Design thinking, Older adults, Ageing in place, Ambient assisted living, User-centered design

## Abstract

**Background:**

Ageing in place is a common desire among older adults and people in need of care. Accessible housing and ambient assisted living (AAL) technologies can help to live independently at home. However, they cannot replace the human support network of informal caregivers, healthcare professionals and social workers. The needs of these stakeholders should be considered and analysed in order to develop user-friendly and acceptable (digital) solutions for ageing in place while supporting human support networks in fulfilling their roles. This paper presents the first step for a comprehensive multi-level needs analysis within the framework of an user-centered design thinking approach.

**Methods:**

Guideline-based interviews were conducted with healthcare professionals, social workers and an informal caregiver to collect data about the needs of older adults as well as people in need of care, and their human support networks.

**Results:**

The call for more information that is easier to find is a common desire of the three groups. There is agreement on system-based communication and orientation problems, the existence of physical and psychological stress exacerbated by a lack of human resources, the desire for personalised care, the need to feel safe and supported in emergencies, and the need for advice and help with administrative tasks. Overall, the needs of one group are closely linked to those of the other.

**Conclusion:**

Stakeholder selection and diversity are decisive for findings about ageing in place. The overlaps between the stakeholders’ needs offer chances and challenges at the same time for the development of user-friendly, acceptable (digital) solutions and products that support ageing in place.

**Supplementary Information:**

The online version contains supplementary material available at 10.1186/s12877-024-04722-x.

## Background

In 2019, 55.1% of women and 39.6% of men in the German population aged 85–89 required long-term care [1]. With an anticipated 22% increase in the number of people aged over 67 years old by 2035 [2], commonly referred to as the “greying of the globe” [3], it is evident that the population’s need for care will rise dramatically in the coming decades [4]. Taking into account age-related changes, such as physical, functional, psychological and social aspects of life [5], sociodemographic change implies a rising number of chronic diseases and older adults being affected by frailty [6, 7].

The majority of older adults and people in need of care prefer to stay in their homes. The term “ageing in place” describes this common desire [8, 9]. The World Health Organization (WHO) defines ageing in place as “meeting the desire and ability of people, through the provision of appropriate services and assistance, to remain living relatively independently in the community in his or her current home or an appropriate level of housing. Ageing in place aims to prevent or delay more traumatic moves to dependent facilities, such as a nursing home” [10]. This study focuses on older adults. However, the concept of ageing in place is also relevant to other groups in need of care and support, such as people with disabilities, who will also be considered for reasons of field characteristics. At present, approximately 56% of people in need of care already have the support of informal caregivers at home [11]. Informal caregivers are people who provide unpaid care to people with whom they have a social relationship (e.g. family, friends, neighbours) for an extended period. This care may take the form of domestic, physical or psychosocial assistance [12–14]. As a result, ageing in place preferences turn the private home into a workplace for healthcare professionals [15]. Therefore, new housing and support concepts need to become part of the provision of care to empower and help older adults and people in need of care to live at home for longer, while conserving human resources, such as medical staff, informal caregivers, and volunteers [15, 16].

Simultaneously, recent discussions and applications of Ambient Assisted Living (AAL) reflect the ongoing shift in care structures. AAL solutions are being developed to support informal caregivers and healthcare professionals who are overburdened as well as people who like to age in place [17]. In fact, older adults are documented to see AAL solutions as a way to reduce the strain on informal caregivers [18]. AAL solutions may include smart home technologies, information and communication technologies, video games, medication reminders, and wearables [9, 19, 20]. In the following, the term AAL refers to concepts, products and services that incorporate new technologies with elements of social interactions aimed at improving people’s quality of life at all stages [21]. Although they are often designed to meet user-specific needs, recent technology-enabled solutions for ageing in place, including AAL, have been criticized for failing to take usability fully into account. The needs of all stakeholders, user acceptance, and user-centered design approaches are not adequately addressed [3, 22, 23]. The development of AAL is often determined by the availability of technologies rather than the users’ needs [18, 24].

In this context, technology acceptance studies confirm that users perceptions of usefulness and ease of use are decisive [25]. Further significant stakeholders are informal caregivers, healthcare professionals and social workers [26] as they influence older adults’ acceptance of AAL technologies in an unprecedented way [27–29]. Moreover, the lack of stakeholder involvement is known as a barrier to technology adoption. An understanding of the stakeholders needs is needed to develop and implement AAL technologies [27, 30]. In summary, the stakeholders’ training with and knowledge of AAL technologies are critical factors for adoption decisions and successful implementation [27]. Given the influence of informal caregivers and healthcare professionals on the acceptance of AAL technologies and as potential primary users, studies suggest that the perspectives of both informal and formal caregivers should be considered [18, 31]. Consequently, the identification of stakeholders’ needs is a prerequisite for developing user-friendly and easy-to-implement AAL solutions [27].

Studies show that the needs of older adults and people in need of care are primarily determined by health status and social embedding [32]. Recommendations for future research include understanding how older adults cope with social problems, identifying the support needed by older adults to manage multimorbidity, determining the most effective way to address psychological needs, and understanding the care and support needs of other stakeholders, such as informal caregivers and healthcare professionals, particularly those who are older. Research on diversity in health stresses the significance of the perspectives of those affected and their diversity. Differences exist in how older adults’ handle social problems and the support they require to manage multimorbidity [33]. Stakeholders are particularly likely to employ solutions that meet older adults’ needs in the long-term [27]. Therefore, a needs assessment for ageing in place solutions must take into account the multiple perspectives in the field.

For developing user-centered AAL innovations, design thinking is a promising approach [34]. It is an iterative, interdisciplinary, participatory process that involves several rounds of idea generation, prototyping, and testing, with each step focusing on the users’ needs. Studies confirm that new healthcare services and solutions developed using design thinking and other user-centered methods are more user-friendly, effective, and more widely accepted than those interventions developed by using more traditional methods [35]. In design thinking, the first and most important step involves analyzing and understanding the problems and needs of all relevant stakeholders to establish empathy [36]. On this basis, solutions are then designed to address users’ needs. According to the Hasso - Plattner Institute, design thinking processes can be divided into six stages [37]. The first three stages, which can be referred to as exploration of the problem, are understanding, empathizing, and synthesizing. The stages ideation, prototyping, and testing explore the solution [37]. This paper illustrates the procedure and results of the first three stages of a design thinking process applied to assess stakeholders’ needs for developing ageing in place solutions.

In the research project DeinHaus 4.0 - Oberbayern older adults, post-rehab patients, and people with disabilities and other care needs are informed about assistive devices for independent living at home. In order to equip model residences with assistive design furniture and AAL technologies in a user-centered and user-friendly way, a needs assessment was conducted using the design thinking method.

### Research objective

To understand the needs of stakeholders who play a significant role in implementing and using new digital solutions [38], guideline-based interviews were conducted with stakeholders from the health and social care sector: Healthcare professionals, social workers, and informal caregivers involved in home care.

### Project description

**Fig. 1:**
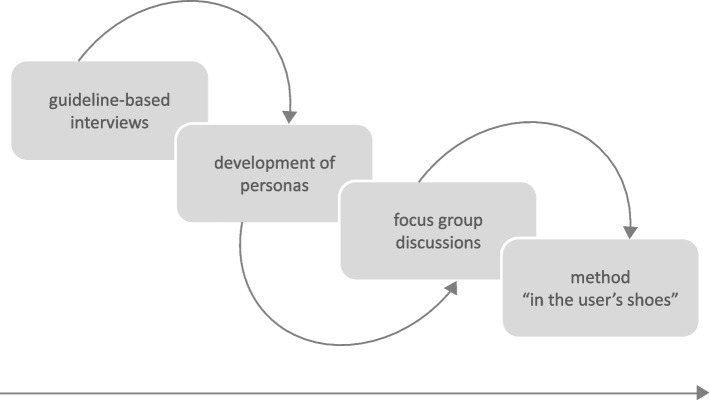
Components of a design thinking-based multilevel needs analysis

The research project DeinHaus 4.0 – Oberbayern, aims to inform older adults, post-rehab patients, and people with disabilities and other care needs about assistive devices for independent living at home. A needs assessment was conducted using the design thinking method to inform the equipment of model residences with assistive design furniture and AAL technologies. Those AAL technologies, such as smart home technologies (light, temperature, security), sensors for presence and fall detection, wearables for vital signs and activity tracking and technologies to simplify communication, are permanently deployed for the duration of the project in the model residences, that are rented for this purpose. The needs of stakeholders are identified through a multilevel process of design thinking (see Fig. 1). This paper focuses on the first two levels in particular that correspond to the first three stages of a design thinking process.

## Methods

The first step of the needs analysis was to collect data on the perspectives of stakeholders from the social and healthcare sector and those of informal caregivers by means of ten semi-structured, guideline-based stakeholder interviews. Prior to the research, the participants were informed about the aim of the research project and the purpose of the interviews. The data collection procedure was confirmed by a positive ethics vote of the Joint Ethics Committee of the Bavarian Universities of Applied Sciences (GEHBa-202,104-V-022). The interviews were analyzed using qualitative content analysis according to Mayring [39]. The interviews were conducted by the first author, who is female, has a background in physiotherapy and healthcare management, is a PhD student and works as a research assistant in the research project DeinHaus 4.0 – Oberbayern.

### Participant selection

To identify the inclusion criteria for participants in the interviews, a workshop was held with two housing counsellors, who provided insight into the everyday life of the target group. In Germany, housing counsellors are deployed for a region to provide information for the population adapting homes to help people live independently for as long as possible. This includes both older adults and other people in need of care. The expertise of the housing counsellors was used to identify regional organizations and groups of home-based care stakeholders and their representatives. In this way, participants from the social and healthcare sector, the care environment of older adults, such as social and medical services providers, and those in the social environment, such as informal caregivers, were purposively selected. The network of the DeinHaus 4.0 - Oberbayern project, consisting of cooperation partners from the districts of Rosenheim, Berchtesgadener Land and Mühldorf am Inn, was used for the recruitment. The following inclusion criteria were used to select the participants:

- The experts were in direct contact with the people they provide care for.
- The experts were part of the care process for the older adults or people in need of care.
- The experts were accessible via telephone or video communication.
- The experts spoke German or English.

Based on the information of the housing counselors, twenty potential experts were initially contacted by e-mail. Fifteen experts agreed to be interviewed, and in the end, eleven interviews took place. Overall, the experts were an interdisciplinary group with informal caregivers and representatives from the fields of social work, outpatient and inpatient care, medicine and disability care, and rehabilitation (Table 1). Those who gave their written consent to participate were informed by telephone or video platform about the research project DeinHaus 4.0 - Oberbayern. The participants preferred the interviews to be held in German rather than English. Ten interviews were included in the stakeholder needs assessment presented as one interview with a pneumologist was not included because the interviewer’s questions were not answered, but intention and design of the research project was being criticized. Out of the ten interviews included, nine interviews were individual interviews and one interview was conducted with two experts.

**Table 1:** Participant characteristics

Profession	Gender	Age Group	Employing Institution	Role description	Group of affected persons user population is working with
Social Worker	female	40–49	Rehabilitation Clinic	discharge management of rehab patients	patients of neurological rehabilitation
Nurse	female	40–49	Outpatient Care Service	management of an outpatient care service	older adults and informal caregivers
Informal Caregiver	female	50–59	No institution (informal caregiver)	mother taking care of her adult, disabled daughter	young patient with disability
Physician	male	60–69	Rehabilitation Clinic	physician for geriatric rehabilitation	older adults with frailty
Pedagogue	male	60–69	Counselling Centre (for Open Disability Work)	divisional management handicapped assistance	people with mental disabilities
Social Worker	female	50–59	Counselling Centre	consulting and assistance	people with acquired brain injury
Social pedagogue	female	50–59	Counselling Centre	consulting and assistance	informal caregivers
Nurse	male	60–69	Outpatient Care Service	management of an outpatient care service	older adults and informal caregivers
Nurse	female	50–59	Outpatient Care Service	nurse in an outpatient care service	older adults and informal caregivers
Lawyer	male	50–59	Outpatient Care Service & Nursing Home	managing director of outpatient and inpatient nursing service	mainly employees (nurses, social workers)
Nurse	female	70–79	Retired (Nursing Home), Volunteer Service	gerontopsychiatric specialist, senior citizen work	older adults

### Data collection

The semi-structured interview guideline (see Additional file 1) was developed by the team of the research project DeinHaus 4.0 – Oberbayern based on a literature review. The interviews started with a brief introduction including the name and occupation of the interviewer, followed by an opening question about the participants’ (professional) care-related roles and their relationships with the people concerned. The introduction was followed by open-ended questions about the needs of the care recipients, the components of the care process, the potential and challenges of integrating AAL and smart home technologies into the care process, and the challenges of applying AAL technologies in the home environment. Regarding participants’ roles, it cannot be excluded that the participants had different levels of knowledge about AAL technologies. The interviews lasted between 30 and 60 minutes and were documented by audio recording. No field notes were taken. The interviews were conducted under contact restrictions due to the COVID-19 pandemic. They were held via video communication platforms or by telephone. Video communication made it easier for the interviewers to guide the interviews, as facial expressions and gestures helped to formulate follow-up questions. However, one interview was conducted by telephone for reasons of participant preferences. The transcripts were then sent to the interviewees for reasons of transparency and data privacy protection. No repeat interviews were carried out.

### Data analysis

The audio recordings of the interviews were fully transcribed using f4 transcription software, following Kuckartz’s transcription rules for computer-assisted evaluation including fourteen rules, such as a literal transcription and a slight smoothing of language and punctuation [40]. The material was analysed using Mayring’s summary rules of interpretation [39]. Therefore, the stakeholders’ paraphrased and abridged statements were coded using inductive category formation [39]. Previously coded transcripts were re-examined in an iterative process as new codes emerged. MAXQDA analysis software was used for data analysis in all steps [41]. The code systems were merged into categories. They were validated through interpersonal consensus building among researchers involved in the DeinHaus 4.0-Oberbayern project with backgrounds in physiotherapy, nursing or psychology [42]. Qualitative studies have shown, that most new codes typically emerge between six and twelve interviews. Data saturation often occurs after 12 interviews [43, 44]. In the analysis presented, data saturation occurred after 10 interviews. The project team reflected on the codes and interpretations in a workshop. All categories are presented in Table 2. The quotes in the results section that follows were translated into English.

**Table 2:**
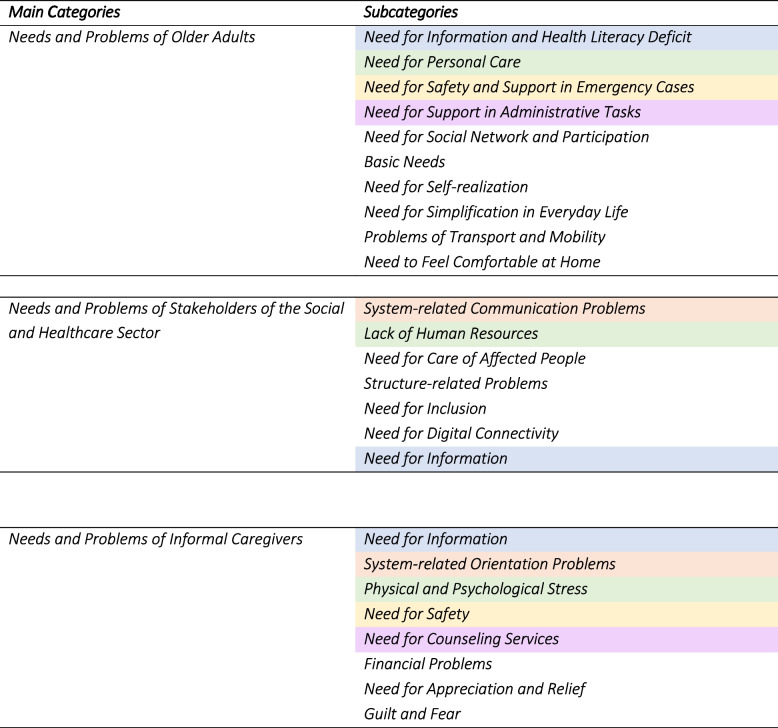
Overview of categories of the stakeholder interviews

## Results

The main categories are the needs of older adults, informal caregivers and healthcare professionals. The results section refers to ‘older adults’ as this is the group with whom most of the interviewed experts work. However, we also refer to other groups such as people with disabilities and those with care and support needs. When comparing the subcategories specific to each group, it became evident that participants assigned similar or closely related issues and requirements to different groups. The similar or closely related needs of the three groups (e.g. information deficits and lack of health literacy) are highlighted in same color in Table 2 and explained in more detail below. Most of related, overlapping categories were identified for the informal caregivers. However, the participants also identified group-specific categories of needs that do not overlap or are not related to the needs of other groups. These categories are also listed in Table 2, but are not highlighted in colour.

### Needs and problems of older adults

The stakeholders emphasise the need for support of older adults: in emergencies, in administration, in navigating the German healthcare system and in personalised care.

#### Health literacy deficiencies in older adults

The experts highlighted a contradiction in the German healthcare system, as it is designed for active, responsible patients. At the same time, many patients, particularly older adults without social support, struggle while attempting to navigate the system.

*“Everything is actually geared toward the responsible patient who gets information and consciously makes decisions that are sufficient, ideally after they have informed themselves beforehand. And that is a situation with which many patients are extremely overburdened and which is also not well implemented in the system.”* (Transcript 1, Section 15).

A lack of access to available health and social services makes it difficult for those in need of care to navigate the healthcare system. In contrast, people with higher levels of education and technical knowledge are better equipped to take advantage of the healthcare system and its technological opportunities.

#### Need for personal care of older adults

The experts emphasized the wish of older adults for personalized care. While digital and technical solutions can be helpful, they cannot fully meet these needs. Those affected also value consistency in long-term medical caregiving. Older adults prefer to be treated by the same general practitioner, particularly if they have to move to a nursing home.

*“I believe that the digital will not be able to completely replace the analog. And you can see that, especially we humans simply need the encounter.”* (Transcript 8, Section 38).

The experts also mentioned specific tasks that require personal and medical care. These include pressure ulcer prophylaxis and pain relief, which are crucial for severely affected patients. Many patients may already find it challenging to manage their prescription medication and measure their vital signs.

#### Need for safety and support in emergencies for older adults

*“So interaction with the outside world is often an issue. So many, many patients have the issue of providing for safety. That means that maybe they live alone and there’s a risk that if they fall, nobody notices for a very long time.”* (Transcript 1, Section 12).

A primary concern for older adults is safety. This group has a particular fear of falling and left being unattended. In this case, the experts recommend home emergency systems to provide security. Additionally, there is a need for older adults financial security when inpatient care is the only option.

#### Need for support for administrative tasks for older adults

The experts observed that many older adults feel overwhelmed by administrative tasks, particularly when communicating with health institutions and insurance companies. These challenges are often the result of difficulties with written communication. Older adults may have limited knowledge of who to contact for help, their rights, and how to make claims, such as with a health insurance fund. After rehabilitation stays, older adults may feel overburdened. They require assistance in establishing new daily routines and organising of therapies and medications.

### Needs and problems of informal caregivers

Informal caregivers are reported to need information and orientation, alleviation of stress and confirmation of safety.

#### Informal caregivers’ need for information

Informal caregivers often face problems with organizational tasks, such as applying for care levels and making enquiries for medical aid and support services (e.g., household-related services, group offers, day and night care, and short-term care). Mainly informal caregivers are either unaware that they are entitled to receive help or unaware of how to claim it.

*“ So if I am a family caregiver or if I want to know what my partner in need of care is entitled to in terms of claims against the state, health insurance funds, and long-term care insurance funds, there are huge gaps in my knowledge.”* (Transcript 6, Section 17).

Thus there is a need for information, support in financial and legal matters, and counseling services for informal caregivers to provide care for family members in a home setting.

#### System-related orientation problems of informal caregivers

The authors defined system-related orientation problems as perceived issues that arise from interactions with healthcare institutions for those affected.

Institutions in the healthcare system do not provide sufficient support for financing medical aid or planning annual budgets. Informal caregivers often have to appeal to insurance companies to obtain funding for medical equipment, but many lack the resources to do so. The related barriers to medical aid are even greater for non-German-speaking informal caregivers.

#### Physical and psychological stress of informal caregivers

*“People are simply overwhelmed and no longer know how to organize their care, and how they can obtain relief, even to the point of exceptional situations: What do I do? I have to go to hospital myself, and my relative is not cared for?”* (Transcript 8, Section 10).

Many informal caregivers are overburdened with caring for their relatives. Becoming an informal caregiver often occurs suddenly and may be linked to a relative’s hospitalization. However, issues can arise after the patient is discharged. Informal caregivers are often unprepared for the new care situation, and the home may not be adequately set up for it. Discharges from clinics, often occur despite a significant need for care, leading to difficult situations for both informal caregivers and those being cared for. Experts describe being an informal caregiver as involving a high level of physical and mental strain, especially for caregivers aged 65 years and older, which is often the case with spouses or in relatives of people with disabilities. To support informal caregivers, new solutions need to provide psychosocial support to enable them to have time for themselves outside of caregiving.

#### Informal caregivers’ need for safety

It is crucial for informal caregivers to have the assurance that their relatives are safe, especially when they are alone at home or outside on their own. Experts have noted a high level of acceptance among informal caregivers for technical solutions that ensure the safety of those being cared for.

#### Informal caregivers’ need for counselling services

*“Even more important than the technology would be the support of the people, so that they have the feeling that they don’t always have to fight against something, but that it is also [...]. It’s not about appreciation, it’s about being able to get what you’re entitled to in the system.”* (Transcript 4, Section 117).

The experts noted a lack of counselling services, especially for people with disabilities and their informal caregivers, in coping with care-related bureaucratic tasks. Similarly, there is a shortage of counselling services to relieve the burden on informal caregivers, who often need to be persuaded to accept support. The experts also highlighted that older adults and their informal caregivers are often unaware of where to seek help and advice. Social services, which are often based in hospitals, are essential for providing advice and assistance in preparation for discharge to a home setting. Some services, such as domestic support and care services, can alleviate the burden on informal caregivers and may act as door openers.

### Needs and problems of stakeholders of the social and healthcare sector

Information needs, lack of communication between health care institutions and lack of human resources are criticised by stakeholders.

#### Social and healthcare sector experts’ need for information

The experts requested additional opportunities to learn about AAL, in order to provide recommendations to informal caregivers and older adults. To overcome barriers to the utilization of AAL, the experts call for readily accessible and easy-to-understand information, such as video-based explanations, for people in need of care or support.

In addition to older adults, young people may be an important target group for information on accessible buildings, without which AAL and smart home solutions cannot enable people to live independently in their homes.

#### System-related communication problems of experts in the social and healthcare sector

This quote illustrates the contradiction in healthcare systems that favor active patients, despite many patients are being passive.

*“And that is a very big problem, because many people put themselves in the role of the passive patient and don’t see or use many possibilities to shape things for themselves and see themselves in a role that is actually no longer directly intended by our health system.”* (Transcript 1, Section 15).

According to the experts, another systemic problem is the lack of communication among different providers (e.g. physicians and pharmacies), which hinders joint access, as is the division between health and long-term care insurance. Furthermore, parallel structures in the care sector, particularly in the areas of training and education, prevent experts from gaining a clear understanding of those different structures.

The lack of communication systems among providers is, in part linked to strict data protection regulations. While being particularly important for personal health data, they can slow down the digitalization in the healthcare system. For instance, previously popular communication channels cannot be used for communication and data transfers among healthcare professionals.

#### Lack of human resources for experts in the social and healthcare sector

*“But we all know that the developments are such that there will be more and more older people in need of help and, at the same time fewer and fewer caregivers. It is already precarious. And then it makes sense to consider what can be digitized, particularly in terms of security and continuous support, supplemented by personal support.* “(Transcript 8, Section 38).

The social and healthcare sector faces challenges due to a lack of human resources, specifically nursing staff shortages, and the expected sociodemographic change. The nursing profession experiences significant physical and mental strain, especially, in the inpatient sector. For example, nurses do not have breaks to rest between caring for different patients. To conserve healthcare workers resources, certain tasks should be delegated non-healthcare workers and technological solutions, keeping in mind that the resources for professional care will remain limited in the future.

### Overlapping needs

Comparing the needs categories, it becomes apparent that the needs of the three groups overlap in certain respects. A common desire of the three groups is the call for more information that is easier to find. There is agreement on system-related problems, on the presence of physical and psychological stress exacerbated by a lack of human resources versus desires for personal care, the need for safety and support in emergencies as well as the need for counselling services and support in administrative tasks. Overall, the needs of one group are closely linked to the needs of the other. While older adults wish for personalised care and a personal connection with the caregiver, informal caregivers often feel physically and mentally overwhelmed by the care they provide. Similarly, in the group of healthcare professionals, older adults’ wishes conflict with a shortage of workers and the heavy burden placed on healthcare professionals.

## Discussion

The stakeholder needs analysis identifies group-specific and overlapping needs of older adults, informal caregivers and health and social care professionals. There are information deficits and support needs in administrative tasks, system-related communication and orientation problems, and the nexus of lacking human resources versus personalized care. Considering the yields and limitations of the stakeholder needs assessment, the results are the a base for the research project DeinHaus 4.0 – Oberbayern. As a second step of the multi-level needs analysis, following the design thinking stages of understanding, empathizing and synthesizing, five user personas were developed [3, 45] based on the results. User personas are fictitious user profiles created from target group observations. By means of visualizations, they help to better understand the users’ needs, challenges and motivations for building empathy, which is critical in developing consumer health technologies (CHT) [45]. Both the main categories identified in the interviews (Table 2) and the personas were used as stimuli in focus group discussions aimed at understanding the needs and perspectives of people with care and support needs related to ageing in place supported by AAL systems, taking into account the limitations of the study. These focus group discussions are currently being evaluated, and a design thinking method called “in the user’s shoes” is being prepared [46].

### Group-specific needs

A larger number of needs that overlap with those of the other groups have been identified in the group of informal caregivers, which may be explained by the heavy burden that informal caregivers have to bear and their essential role in providing home care [47]. The need to systematically consider and distinguish between needs of formal and informal caregivers in technology development has been highlighted in other studies [18]. As the needs of informal caregivers were most frequently mentioned in this study, the group may be of particular relevance for technology development and implementation in home care.

### Information deficits and support needs in administrative tasks

There is a basic need for more information on healthcare-related topics, the home care of older adults in particular. The lack of knowledge about the support available in the German healthcare system is a known problem for informal caregivers, older adults, and people requiring care [48]. Older adults, in particular, have been shown to need more information about their medical conditions as well as more comprehensive explanations of diagnoses, treatment options and medications [49]. Both older adults and informal caregivers are overwhelmed with the communication involved in applying to relevant institutions for financial support or reimbursement. Due to the lack of information, family members and informal caregivers feel that they have to advocate for and obtain medical and service information [49]. At the same time, informal caregivers need support in obtaining financial support and authorization for medical equipment. Obstacles to accessing health insurance payments were described. The lack of information described by social and healthcare stakeholders is the need for learning materials on AAL and smart home systems to inform their patients about the latest developments in digital and technological aids to daily living.

The stakeholder needs assessment identifies tasks, especially of older adults and informal caregivers, that have not yet been listed as areas of technology application for ageing in place. To the well-known areas of mobility, information and communication technology, biotechnology and ambient intelligence [50], the study at hand adds organizational and administrative tasks. In line with recommendations that technologies should complement face-to-face contact, not replace it [31], the stakeholder needs assessment points to a demand for information transfer and communication on ageing in place that could be met by technology. With older adults preferring counselling services and personal contacts to guide them through the system (e.g. support groups) [51], a gap between technical development possibilities and the needs of the people affected shows up. The stakeholders objected that the development of such tools should focus on simplifying communication and access to information, e.g. about the support opportunities of the social system.

### System-related communication and orientation problems

Several needs identified are rooted in the German healthcare system. At a political level, health literacy should be promoted and included in general education, if patients are to play the active role in the healthcare system. Information (e.g. on support services or financing) must be easy to find and provided in an accessible and inclusive way. The navigation needs documented for older adults are confirmed by the findings about particular difficulties of vulnerable groups in navigating the German health care system. Studies recommend that the structure of the system should be questioned [52].

The lack of communication structures and the challenges of German data protection requirements among various medical providers described by the interviewees are in line with the problems identified by a study on the interdisciplinary treatment of Parkinson’s patients [48]. Digital solutions like AAL can only improve the communication structures, if they meet the requirements above.

### Lack of human resources versus need for personal care

In line with scientific expectations about future trends, the stakeholders agree that more and more older adults are seeking personal care as it is often the only possible social contact for older adults and informal caregivers [33]. This may be the reason for the preferences to be cared for by the same person, that are expected to increase in the future. Studies confirm the physical and mental overload of informal caregivers [53]. In accordance to existing studies calling for problem-related solutions to be more effective in the long term [54, 55], the stakeholders recommend supporting informal caregivers with help services and respite, and identifying which tasks can be performed by non-healthcare workers or solved by technological solutions. According to previous studies, technology can support the creation of meaningful social relationships [31], and is also desirable in professional care settings.

### Need for safety and support in emergencies

Consistent with previous studies identifying security and independence as drivers of the use of AAL by older adults and informal caregivers [23], the stakeholders describe a strong need to feel safe and to know about support options in an emergency. According to the literature, older adults are not only at an especially high risk [56], but also fear of falling is high among fallers and non-fallers [57]. Using the fall risks argument, the stakeholders expect new safety tools to detect or prevent falls to be accepted by older adults.

In sum, the needs voiced by the stakeholders confirm that new digital solutions have the potential to simplify complex situations in the provision of healthcare services but the areas of application are not clearly defined yet. There are limitations with regard to personal interactions.

### Limitations

The present study has limitations in terms of space, sample size and subjectivity in data analysis. The sampling strategy allows for a diverse picture of older adults’ needs in home care. The small sample size (*n* = 11) included stakeholders with differing professional backgrounds and from a variety of health care institutions. Thus, it cannot be excluded that participants have different levels of knowledge about AAL. With the participant selection focusing on the region, reflections on the diversity of older adults, informal caregivers and stakeholders are the next step necessary to cover the range of needs and their overlaps. When interpreting the results, it is important to take the region’s rural character and the relative affluency of the population into account. Cultural differences, which may influence perceptions of loneliness in ageing [58] and cultures of (health) care, need to be considered when transferring the results to regions and countries.

Future studies should focus on understanding the needs of older adults and beyond, in particular involving these groups in the co-creative design and development of AAL. Consideration should be given to how AAL solutions could meet the changing needs and health status including cognitive impairment of people in need of care and informal caregivers over time, as well as the subjective potential disadvantages or unintended consequences of AAL [29]. This study focuses on the needs of stakeholders primarily involved in the care of older adults. In the future, there is a need to systematically include the diverse group of stakeholders, responsible for the care of other groups, such as young people and people with disabilities. However, other groups also need to be consulted in order to progress the implementation of AAL. Future research has to concentrate on the identification and the implementation of solutions, based on, for example, interviews with representatives of health and long-term care insurance funds, and staff of political and legal entities.

## Conclusion

The stakeholder needs assessment at hand shows that different groups have intersecting needs and problems – a condition that helps addressing the challenges. These intersections offer an opportunity to address the needs of different groups at the same time, but also present a challenge to meet the needs of all of them. For example, creating technological solutions that reduce the burden on informal caregivers but don’t reduce the personal contact older adults want. Further, the intersections are evident within the following categories of needs: “information deficits and lack of health literacy“, “system-related communication and orientation problems”, “physical and psychological stress and lack of human resources versus need for personal care”, “need for safety and support in emergencies” and, „need for counseling services and support for administrative tasks”. The intersections demonstrate the importance of including all stakeholders in the development and implementation of easy-to-use, acceptable (digital) solutions for ageing in place and pending improvements within the health care system.

### Supplementary Information

**Additional file 1. **

## Data Availability

The datasets generated and analyzed during the current study can be made available from the corresponding author on reasonable request and under a data sharing agreement.

## Abbreviations

RKI: Robert Koch Institut
WHO: World Health Organization
AAL: Ambient Assisted Living
ICF: International Classification of Functioning, Disability and Health
CHT: Consumer Health Technologies
