# Supplementary material for: Stakeholder needs assessment for developing ageing in place solutions – a qualitative study
Source: BMC Geriatr. 2024 Jan 29;24:104. doi: 10.1186/s12877-024-04722-x (PMC10823612; doi:10.1186/s12877-024-04722-x)
Supplement: Supplementary file 1 — Additional file 1. [file 12877_2024_4722_MOESM1_ESM.pdf]

Interview Guideline

Profession: Date:

Contextual Questions

1. In what role do you work with the stakeholders (persons affected)?
2. With which groups of stakeholders do you work?

Questions addressing needs

3. What problems/ issues do people express or do you identify with them?
   a) What are the five most important problems?/ How would you prioritize the problems?
4. What needs do people express in the care process?
   a) How would you prioritise the needs?
   b) Can you think of any other needs?

Process questions

5. Which components (e.g. outpatient specialist care, outpatient nursing service, general practitioner care or physiotherapy) of the care process are, in your view particularly important for the long-term success after rehabilitation?
   a) Which target group did you have in mind when answering this question?

6. What chances do you see for the care process, e.g. in an outpatient rehabilitation concept based on AAL (technical assistance) systems (e.g. fall detectors, daily living aids), according to your experience?

7. How can concepts with AAL systems and smart home facilities secure the successes achieved and the ability to work in the long term, according to your experience?

Acceptance questions

8. What challenges do you see with regard to the application of AAL/Smart Home systems in the home environment?

a) ...in the implementation phase?

b) ...in the long-term use?

c) Are there any risks that you associate with the application of AAL/ Smart Home?

Final question

9. What opportunities/potentials in terms of treatment/rehab success do you see in the application of concepts based on digital and analogue solutions?

a) Using the example of AAL// Smart Home solutions (sensors, technical aids)
b) Using the example of analogue solutions (outpatient care services, neighbourhood help, self-help groups, housing counsellors, etc.)
